# Effect of dialysis modality on frailty phenotype, disability, and health-related quality of life in maintenance dialysis patients

**DOI:** 10.1371/journal.pone.0176814

**Published:** 2017-05-03

**Authors:** Seok Hui Kang, Jun Young Do, So-Young Lee, Jun Chul Kim

**Affiliations:** 1 Division of Nephrology, Department of Internal Medicine, Yeungnam University Hospital, Daegu, Republic of Korea; 2 Division of Nephrology, Department of Internal Medicine, CHA Bundang Medical Center, CHA University School of Medicine, Seongnam, Republic of Korea; 3 Division of Nephrology, Department of Internal Medicine, CHA Gumi Medical Center, CHA University, Gumi, Republic of Korea; Hospital Universitario de la Princesa, SPAIN

## Abstract

**Background:**

Health-related quality of life (HRQoL) surveys are needed to evaluate regional and ethnic specificies. The aim of the present study was to evaluate the differences in HRQoL, frailty, and disability according to dialysis modality in the Korean population.

**Patients and methods:**

We enrolled relatively stable maintenance dialysis patients. A total of 1,616 patients were recruited into our study. The demographic and laboratory data collected at enrollment included age, sex, comorbidities, frailty, disability, and HRQoL scales.

**Results:**

A total of 1,250 and 366 participants underwent hemodialysis (HD) and peritoneal dialysis (PD), respectively. The numbers of participants with pre-frailty and frailty were 578 (46.2%) and 422 (33.8%) in HD patients, and 165 (45.1%) and 137 (37.4%) in PD patients, respectively (*P* = 0.349). Participants with a disability included 195 (15.6%) HD patients and 109 (29.8%) PD patients (*P* < 0.001). On multivariate analysis, the mean physical component scale (PCS) and mental component scale (MCS), symptom/problems, and sleep scores were higher in HD patients than in PD patients. Cox regression analyses showed that an increased PCS in both HD and PD patients was positively associated with patient survival and first hospitalization–free survival. An increased MCS in both HD and PD patients was positively associated with first hospitalization–free survival only.

**Conclusion:**

There was no significant difference in frailty between patients treated with the two dialysis modalities; however, disability was more common in PD patients than in HD patients. The MCS and PCS were more favorable in HD patients than in PD patients. Symptom/problems, sleep, quality of social interaction, and social support were more favorable in HD patients than in PD patients; however, patient satisfaction and dialysis staff encouragement were more favorable in PD patients than in HD patients.

## Background

Chronic kidney disease is a well-known public health problem that can progress to end-stage renal disease (ESRD), which requires renal replacement therapies such as kidney transplantation, hemodialysis (HD), and peritoneal dialysis (PD). The prevalence of ESRD is approximately 2,034 per million in the US population and 1,571.5 per million in the Korea population [1,2]. Although many interventions can prevent the progression to ESRD, cases of ESRD continue to increase over time, a phenomenon that will continue with increased life expectancy and comorbidities such as diabetes mellitus (DM) and hypertension. Kidney transplantation is the ideal method for treating ESRD patients; however, a lack of kidney donors is the main hurdle for this method. Of all ESRD patients, 70.8% were receiving HD or PD [1].

Frailty is a clinical syndrome that was originally defined by gerontologists to describe cumulative declines across multiple physiological systems [3,4]. However, ESRD patients are inherently at a higher risk of insulin resistance, malnutrition, and inflammation than the general population [5]. These conditions can induce the early development and high prevalence of frailty in dialysis patients. Recent studies have focused on the importance of frailty in dialysis patients; however, few studies have examined the differences in frailty according to dialysis modality [6–8].

The health-related quality of life (HRQoL) of dialysis patients is lower than that of the general population or patients who undergo kidney transplantation, and a low HRQoL is associated with decreased survival and more frequent hospitalization in dialysis patients [9–13]. Proper evaluation of and intervention for HRQoL are important for improving prognosis in dialysis patients. However, there are conflicting results about the association between HRQoL and dialysis modality [10,14–17]. Regional and national disparities may lead researchers to various conclusions concerning the association between HRQoL and dialysis modality. Therefore, HRQoL surveys are needed to evaluate regional and ethnic specificies. Although previous studies have investigated the association between HRQoL and dialysis modality in ESRD patients, few have demonstrated the association between HRQoL and dialysis modality in the Korean populations. The aim of the present study was to evaluate the differences in HRQoL, frailty, and disability according to dialysis modality in the Korean population.

## Patients and methods

### Study population

The study participants were those enrolled in a previous study [18]. Briefly, the study participants were recruited from 27 hospitals or dialysis centers in Daegu/Kyungsangpook-do between July and December 2012. A total of 2,737 participants who had undergone HD or PD were included. Among these patients, 1,079 were excluded for being <20 years old (n = 12), receiving dialysis for <6 months (n = 164), having a history of hospitalization in the last 3 months except for vascular access problem in HD patients (n = 351), being unable to walk with or without an assistive device (n = 79), being unable to communicate with the interviewer (n = 149), refusing to provide informed consent (n = 254), or not having laboratory findings (n = 112). A total of 1,616 patients were recruited into our study. This study was approved by the institutional review board of Yeungnam University Hospital (2016-06-022).

### Study variables

The demographic and laboratory data collected at enrollment included age, sex, body mass index (BMI; kg/m^2^), DM status, coronary artery disease (CAD), cerebrovascular disease (CVD), dialysis duration (years), dialysis center (tertiary medical center or not), education level, HRQoL scale scores, and hemoglobin (mg/dL), serum albumin (mg/dL), blood urea nitrogen (BUN, mg/dL), creatinine (mg/dL), calcium (mg/dL), phosphorus (mg/dL), total cholesterol (mg/dL), and intact-parathyroid hormone (i-PTH, pg/mL) levels. Serum albumin, BUN, creatinine, calcium, phosphorus, and total cholesterol levels were measured monthly. The values for the 3 months prior to enrollment were averaged. BUN samples were drawn at the midweek treatment. DM was defined as a self-reported history and medical record of DM diagnosis, or a fasting glucose level of ≥126 mg/dL. CAD was defined as a self-reported history and medical record of angina, myocardial infarction, or congestive heart failure. CVD was defined as a self-reported history and medical record of stroke. All mortality and hospitalization events were retrieved from medical records up to December 2014. If a patient with HD was admitted for a vascular access–related problem, the event was not considered a significant hospitalization.

### HRQoL assessment

HRQoL was assessed by using the Kidney Disease Quality of Life-Short Form (KDQOL–SF) 1.3 Korean version [19]. KDQOL–SF 1.3 includes the SF-36 scale (36 items) and a kidney disease–specific scale (11 items). SF-36 includes eight domains–physical functioning (PF), role limitations due to physical health problems (RP), body pain (BP), general health (GH), vitality (VT), social functioning (SF), role limitations due to emotional problems (RE), and mental health (MH)–and the overall health rating (OHR). A total score of 0–100 was calculated for each domain. A low score indicates a low quality of life. These tools were used to calculate two summary scales: a physical component scale (PCS) and a mental component scale (MCS) [20,21]. The 11 kidney disease-specific items consisted of symptom/problems, effects of kidney disease, burden of kidney disease, work status, cognitive function, quality of social interaction, sexual function, sleep, social support, patient satisfaction, and dialysis staff encouragement.

### Frailty phenotype and disability assessments

We evaluated disability by using four questions on activities of daily living (ADLs) concerning whether the participants currently need help from another person in feeding, dressing/undressing, getting in/out of bed, or taking a bath/shower. For each question, they participants provided one of three responses: no help, some help, or full help. Disability was defined as the inability to perform at least one of the four ADL domains with no help [22]. We scored each ADL as requiring no help (0), some help (1), or full help (2). We also defined the total disability score as the sum of the individual ADL scores.

Frailty was defined by using previously described modified criteria [23]. Briefly, the components consisted of slowness/weakness, poor endurance/exhaustion, physical inactivity, and unintentional weight loss. Slowness/weakness and poor endurance/exhaustion were determined by using the PF scale (2 points for PF scale <75) and the VT scale (1 point for VT scale <55) of SF-36, respectively. Physical inactivity was defined as being physically active <1 time per week during leisure time for the last 3 months (1 point for physical inactivity). Unintentional weight loss was defined as an unintentional body weight loss of > 4.5 kg or 5% of the baseline value during the last year (1 point for weight loss). All points of each frailty component were summed. Those participants with ≥ 3 points, 1 or 2 points, and 0 points were defined as having frailty, pre-frailty, and non-frailty, respectively.

### Statistical analyses

The data were analyzed by using the statistical software SPSS version 21 (SPSS Inc., Chicago, IL, USA). Categorical variables are expressed as both counts and percentages. Continuous variables are expressed as mean ± standard deviation or mean ± standard error (SE). Pearson’s χ^2^ or Fisher’s exact test was used to analyze categorical variables. For continuous variables, means were compared by using Student’s *t*-test or analysis of variance, followed by a post-hoc Tukey comparison. A linear regression analysis was performed to assess the independent predictors of the HRQoL scales. The survival estimates were calculated by using Kaplan–Meier with the log-rank test and Cox regression analyses.

Multivariate analysis was performed by using analysis of covariance, multivariate linear regression analysis, or multivariate Cox regression analysis. For the analysis of covariance and linear regression analysis, the covariates were age, sex, BMI, education level, dialysis duration, DM, CVD, CAD, serum albumin level, BUN level, serum creatinine level, i-PTH level, and total cholesterol level. Multivariate Cox regression analyses were performed by using enter mode with adjustment for age, sex, BMI, education level, dialysis duration, DM, CVD, CAD, serum albumin level, BUN level, serum creatinine level, i-PTH level, and total cholesterol level. The level of statistical significance was set at *P* < 0.05.

## Results

### Participants’ clinical characteristics

A total of 1,250 and 366 participants underwent HD and PD, respectively (Table 1). Age and hemoglobin, serum albumin, and BUN levels were higher in HD patients than in PD patients. BMI and serum creatinine, total cholesterol, and i-PTH levels were higher in PD patients than in HD patients. A greater proportion of HD patients than PD patients had DM, CAD, or CVD, whereas the proportion of male sex, the education levels, and the calcium and phosphorus levels were similar between the two groups.

**Table 1:** Participants’ clinical characteristics.

	Hemodialysis (n = 1,250)	Peritoneal dialysis (n = 366)	*P*-value*
Age (years)	56.4 ± 13.2	54.1 ± 11.9	0.003
Male sex (%)	708 (56.6%)	195 (53.3%)	0.255
Body mass index (kg/m^2^)	22.1 ± 3.2	23.5 ± 3.1	<0.001
Diabetes mellitus (%)	516 (41.3%)	123 (33.6%)	0.008
Coronary artery disease (%)	221 (17.7%)	33 (9.0%)	<0.001
Cerebrovascular disease (%)	129 (10.3%)	15 (4.1%)	<0.001
Tertiary center (%)	525 (42.0%)	359 (97.3%)	<0.001
Dialysis vintage (years)	5.1 ± 4.6	5.3 ± 3.9	0.409
Education level			0.121
≤ 6^th^ grade	285 (22.8%)	69 (18.9%)	
7^th^–12^th^ grade	253 (20.2%)	67 (18.3%)	
> 12^th^ grade	712 (57.0%)	230 (62.8%)	
Hemoglobin (mg/dL)	10.5 ± 0.8	10.4 ± 1.1	0.016
Serum albumin (mg/dL)	4.0 ± 0.3	3.6 ± 0.4	<0.001
Blood urea nitrogen (mg/dL)	61.9 ± 14.3	53.5 ± 15.5	<0.001
Creatinine (mg/dL)	10.3 ± 2.8	11.1 ± 3.6	<0.001
Calcium (mg/dL)	8.7 ± 0.8	8.6 ± 0.9	0.060
Phosphorus (mg/dL)	5.3 ± 1.4	5.3 ± 1.3	0.645
Total cholesterol (mg/dL)	148.6 ± 35.3	172.5 ± 38.0	<0.001
Intact parathyroid hormone (pg/mL)	264.9 ± 309.2	319.6 ± 433.9	0.007

Data are expressed as number (percentage) for categorical variables and mean ± standard deviation for continuous variables.

* *P-*values were tested by using Student’s t-test for continuous variables and Pearson’s χ^2^ test or Fisher**’s** exact test for categorical variables.

### Comparison of frailty and disability according to dialysis modality

The numbers of participants with pre-frailty and frailty were 578 (46.2%) and 422 (33.8%) in HD patients, and 165 (45.1%) and 137 (37.4%) in PD patients, respectively (*P* = 0.349). The total frailty score (mean ± SE) was 1.89 ± 0.04 in HD patients and 1.96 ± 0.08 in PD patients (*P* = 0.433). Among individual components, the scores for slowness/weakness, poor endurance/exhaustion, physical inactivity, and unintentional weight loss were 0.67 ± 0.03, 0.66 ± 0.03, 0.45 ± 0.01, and 0.11 ± 0.01 in HD patients and 0.77 ± 0.05, 0.68 ± 0.02, 0.44 ± 0.03, and 0.07 ± 0.01 in PD patients, respectively (*P* = 0.087 for slowness/weakness; *P* = 0.495 for poor endurance/exhaustion; *P* = 0.702 for physical inactivity; *P* = 0.042 for unintentional weight loss). Multivariate analyses showed that the total frailty score was 1.89 ± 0.04 in HD patients and 1.97 ± 0.09 in PD patients (*P* = 0.445). The scores for slowness/weakness, poor endurance/exhaustion, physical inactivity, and unintentional weight loss were 0.67 ± 0.03, 0.66 ± 0.01, 0.45 ± 0.02, and 0.12 ± 0.01 in HD patients and 0.80 ± 0.05, 0.68 ± 0.03, 0.44 ± 0.03, and 0.06 ± 0.02 in PD patients, respectively (*P* = 0.036 for slowness/weakness; *P* = 0.634 for poor endurance/exhaustion; *P* = 0.650 for physical inactivity; *P* = 0.010 for unintentional weight loss). In a comparison with HD patients, multivariate analyses showed that PD patients had an odds ratio of 1.298 (95% confidence interval [CI], 0.936–1.800; *P* = 0.118) for frailty.

Participants with a disability included 195 (15.6%) HD patients and 109 (29.8%) PD patients (*P* < 0.001). The number of disabilities (mean ± SE) was 0.30 ± 0.02 in the HD group and 0.52 ± 0.05 in the PD group (*P* < 0.001). The total disability score was 0.37 ± 0.03 in HD patients and 0.63 ± 0.07 in PD patients (*P* < 0.001). Among individual components, the scores for feeding, dressing/undressing, getting in/out of bed, and taking a bath/shower were 0.07 ± 0.01, 0.07 ± 0.01, 0.07 ± 0.01, and 0.16 ± 0.01 in HD patients and 0.15 ± 0.03, 0.10 ± 0.02, 0.09 ± 0.02, and 0.30 ± 0.03 in PD patients, respectively (*P* = 0.002 for feeding; *P* = 0.216 for dressing/undressing; *P* = 0.312 for getting in/out of bed; *P* < 0.001 for taking a bath/shower). Multivariate analyses showed that the number of disabilities was 0.29 ± 0.03 in the HD group and 0.56 ± 0.05 in the PD group (*P* < 0.001). The total disability score was 0.37 ± 0.04 in HD patients and 0.66 ± 0.07 in PD patients (*P* = 0.001). Among individual components, the scores for feeding, dressing/undressing, getting in/out of bed, and taking a bath/shower were 0.07 ± 0.01, 0.07 ± 0.01, 0.07 ± 0.01, and 0.15 ± 0.01 in HD patients and 0.15 ± 0.03, 0.11 ± 0.02, 0.09 ± 0.02, and 0.32 ± 0.03 in PD patients, respectively (*P* = 0.012 for feeding; *P* = 0.230 for dressing/undressing; *P* = 0.233 for getting in/out of bed; *P* < 0.001 for taking a bath/shower). In a comparison with HD patients, multivariate analyses showed that PD patients had an odds ratio of 3.013 (95% CI, 2.076–4.373; *P* = 0.118) for disability. There was no significant difference in frailty phenotype between HD and PD patients; however, the score for slowness/weakness was higher in PD patients than in HD patients, and the score for unintentional weight loss was higher in HD patients than in PD patients. Disability was more common in PD patients than in HD patients. The scores for feeding and taking a bath/shower were higher in PD patients than in HD patients.

### Comparison of HRQoL scale scores according to dialysis modality

Table 2 shows the differences in HRQoL scale scores according to dialysis modality. On univariate analysis, the mean RE, OHR, PCS, MCS, symptom/problems, and quality of social interaction scores were higher in HD patients than in PD patients, whereas the mean patient satisfaction and dialysis staff encouragement scores were higher in PD patients than in HD patients. On multivariate analysis, the mean PF, GH, SF, RE, OHR, PCS, MCS, symptom/problems, and sleep scores were higher in HD patients than in PD patients.

**Table 2:** Comparison of quality-of-life scales between hemodialysis patients and peritoneal dialysis patients.

	Univariate (mean ± SD)			Multivariate (mean ± SE)		
Short Form-36 scale	HD	PD	*P*-value	HD	PD	*P*-value
PF	74.8 ± 24.1	72.3 ± 22.8	0.082	75.0 ± 0.6	71.6 ± 1.3	0.028
RP	65.7 ± 41.5	61.4 ± 41.1	0.083	66.0 ± 1.2	60.3 ± 2.6	0.060
BP	77.9 ± 25.7	76.1 ± 24.7	0.216	78.1 ± 0.7	75.4 ± 1.6	0.137
GH	45.2 ± 22.9	41.6 ± 20.7	0.008	45.3 ± 0.7	41.1 ± 1.4	0.010
VT	44.8 ± 21.5	44.4 ± 20.4	0.741	44.9 ± 0.7	44.2 ± 1.3	0.646
SF	76.1 ± 28.1	73.1 ± 26.5	0.072	76.6 ± 0.8	71.6 ± 1.7	0.016
RE	72.7 ± 41.1	64.8 ± 43.0	0.001	73.2 ± 1.2	62.9 ± 2.6	0.001
MH	59.1 ± 20.4	58.2 ± 20.4	0.458	59.2 ± 0.6	58.0 ± 1.3	0.418
OHR	38.6 ± 26.3	33.9 ± 24.5	0.003	38.6 ± 0.8	33.9 ± 1.6	0.015
PCS	61.7 ± 20.7	59.2 ± 20.0	0.040	61.9 ± 0.6	58.5 ± 1.2	0.022
MCS	59.6 ± 20.6	56.4 ± 20.0	0.010	59.8 ± 0.6	55.5 ± 1.3	0.004
**KD-specific scale**						
Symptom/problems	80.9 ± 14.5	77.9 ± 16.2	0.001	83.8 ± 0.8	78.8 ± 1.8	0.019
Effects of KD	72.5 ± 19.3	74.6 ± 18.9	0.067	74.6 ± 1.0	76.1 ± 2.3	0.594
Burden of KD	34.6 ± 26.5	36.6 ± 26.2	0.213	39.1 ± 1.5	37.0 ± 3.4	0.591
Work status	27.8 ± 36.0	28.0 ± 37.2	0.939	38.5 ± 2.3	49.1 ± 5.2	0.081
Cognitive function	86.2 ± 17.4	85.4 ± 17.4	0.436	90.3 ± 0.8	86.3 ± 1.9	0.071
Quality of social interaction	76.2 ± 21.8	72.4 ± 21.6	0.003	79.6 ± 1.2	74.4 ± 2.7	0.103
Sexual function	78.0 ± 26.0	75.3 ± 26.7	0.420	78.6 ± 1.5	72.5 ± 3.5	0.131
Sleep	64.2 ± 21.5	62.2 ± 20.9	0.438	68.5 ± 1.2	62.1 ± 2.7	0.044
Social support	68.4 ± 27.0	65.6 ± 27.1	0.080	70.2 ± 1.5	64.6 ± 3.6	0.176
Patient satisfaction	64.6 ± 22.7	70.0 ± 23.0	<0.001	67.3 ± 1.4	66.3 ± 3.1	0.794
DSE	85.1 ± 19.0	90.2 ± 15.9	<0.001	86.8 ± 1.0	89.1 ± 2.4	0.408

*P-*values were tested by using Student’s t-test for univariate analysis and analysis of covariance for multivariate analysis. The multivariate analysis was adjusted for age, sex, body mass index, education level, dialysis duration, diabetes mellitus, cerebrovascular disease, coronary artery disease, serum albumin level, blood urea nitrogen level, serum creatinine level, intact-parathyroid hormone level, and total cholesterol level.

Abbreviations: SD, standard deviation; SE, standard error; HD, hemodialysis; PD, peritoneal dialysis; PF, physical functioning; RP, role limitations due to physical health problems; BP, bodily pain; GH, general health; VT, vitality; SF, social functioning; RE, role limitations due to emotional problems; MH, mental health; OHR, overall health rating; PCS, physical component scale; MCS, mental component scale; KD, kidney disease; DSE, dialysis staff encouragement.

Linear regression analysis showed that, on univariate analysis, PD was inversely associated with GH, RE, OHR, PCS, MCS, symptom/problems, and quality of social interaction**,** and positively associated with patient satisfaction and dialysis staff encouragement (Table 3). Multivariate analysis showed an inverse association between PD and PF, GH, SF, RE, OHR, PCS, MCS, symptom/problems, cognitive function, quality of social interaction, and social support. Positive associations were observed between PD and patient satisfaction or dialysis staff encouragement.

**Table 3:** Linear regression analysis of HRQoL scales in PD and HD patients.

	Univariate		Multivariate	
Short Form-36 scale	β ± SE	*P*-value	β ± SE	*P*-value*
PF	–2.458 ± 1.414	0.082	–3.381 ± 1.540	0.028
RP	–4.261 ± 2.460	0.083	–5.691 ± 3.028	0.060
BP	–1.874 ± 1.515	0.216	–2.754 ± 1.850	0.137
GH	–3.540 ± 1.335	0.008	–4.284 ± 1.651	0.010
VT	–0.417 ± 1.263	0.741	–0.708 ± 1.541	0.646
SF	–2.975 ± 1.651	0.072	–4.945 ± 2.044	0.016
RE	–7.879 ± 2.468	0.001	–10.276 ± 3.061	0.001
MH	–0.901 ± 1.213	0.458	–1.217 ± 1.501	0.418
OHR	–4.665 ± 1.541	0.003	–4.637 ± 1.899	0.015
PCS	–2.510 ± 1.216	0.040	–3.363 ± 1.495	0.022
MCS	–3.142 ± 1.223	0.010	–4.286 ± 1.463	0.004
KD-targeted scale				
Symptom/problems	–2.984 ± 0.885	0.001	–4.104 ± 1.095	0.000
Effects of KD	2.090 ± 1.139	0.067	–0.639 ± 1.408	0.650
Burden of KD	1.954 ± 1.569	0.213	0.018 ± 1.936	0.993
Work status	0.165 ± 2.155	0.939	2.320 ± 2.512	0.356
Cognitive function	–0.806 ± 1.034	0.436	–3.537 ± 1.265	0.005
Quality of social interaction	–3.781 ± 1.292	0.003	–5.116 ± 1.597	0.001
Sexual function	–2.686 ± 3.328	0.420	–6.138 ± 4.085	0.131
Sleep	–0.983 ± 1.269	0.438	–2.660 ± 1.578	0.092
Social support	–2.815 ± 1.607	0.080	–4.789 ± 2.000	0.017
Patient satisfaction	5.464 ± 1.752	0.000	4.322 ± 1.676	0.010
DSE	5.078 ± 1.088	0.000	4.568 ± 1.344	0.001

* A multivariate model analysis was performed by using age, sex, body mass index, education level, dialysis vintage, diabetes mellitus, cerebrovascular disease, coronary artery disease, serum albumin level, blood urea nitrogen level, serum creatinine level, intact-parathyroid hormone level, and total cholesterol level. β was calculated as peritoneal dialysis compared with hemodialysis. Each scale in the HRQoL was a dependent variable.

Abbreviations: HRQoL, health-related quality of life; PD, peritoneal dialysis; HD, hemodialysis; β, unstandardized coefficient; PF, physical functioning; RP, role limitations due to physical health problems; BP, bodily pain; GH, general health; VT, vitality; SF, social functioning; RE, role limitations due to emotional problems; MH, mental health; OHR, overall health rating; PCS, physical component scale; MCS, mental component scale; KD, kidney disease; DSE, dialysis staff encouragement.

Most PD patients were followed at tertiary dialysis centers (97.3%), and we analyzed differences in HRQoL scales according to these groups: tertiary HD patients, non-tertiary HD patients, and tertiary PD patients (S1 Table). Among the three groups, tertiary PD patients had lower mean GH and MCS scores than tertiary HD patients. In addition, tertiary PD patients had the lowest scores for RE, OHR, symptom/problems, and quality of social interaction but the highest scores for dialysis staff encouragement. Tertiary HD patients had the lowest work status scores but the highest social support scores, whereas non-tertiary HD patients had the lowest patient satisfaction scores.

### Clinical outcomes according to HRQoL scales

**Fig 1:**
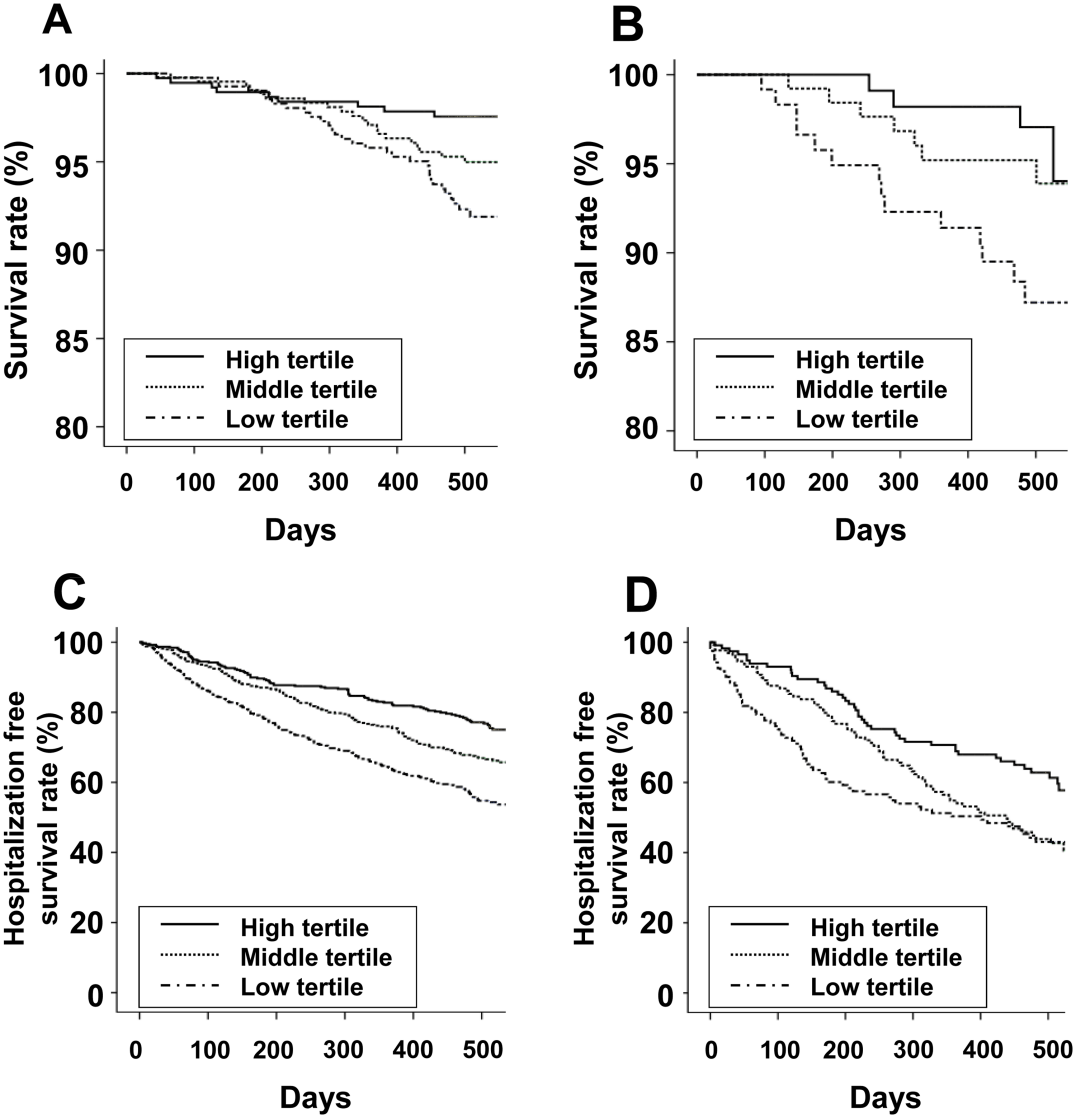
Kaplan-Meier curves of patient survival (A, hemodialysis; B, peritoneal dialysis) and hospitalization-free survival (C, hemodialysis; D, peritoneal dialysis) among the physical component summary score tertiles. (A) Survival rate of hemodialysis patients (High tertile: 99.5% at 100 days and 97.6% at 500 days; Middle tertile: 99.8% at 100 days and 95.0% at 500 days; Low tertile: 99.8% at 100 days and 92.3% at 500 days; *P* = 0.004). (B) Survival rate of peritoneal dialysis patients (High tertile: 100% at 100 days and 97.1% at 500 days; Middle tertile: 100% at 100 days and 95.2% at 500 days; Low tertile: 99.2% at 100 days and 87.2% at 500 days; *P* = 0.024). (C) Hospitalization-free survival rate of hemodialysis patients (High tertile: 94.5% at 100 days and 77.1% at 500 days; Middle tertile: 93.2% at 100 days and 66.9% at 500 days; Low tertile: 86.0% at 100 days and 54.8% at 500 days; *P* < 0.001). (D) Hospitalization-free survival rate of peritoneal dialysis patients (High tertile: 93.0% at 100 days and 62.8% at 500 days; Middle tertile: 87.6% at 100 days and 43.9% at 500 days; Low tertile: 75.2% at 100 days and 43.1% at 500 days; *P* = 0.003).

**Fig 2:**
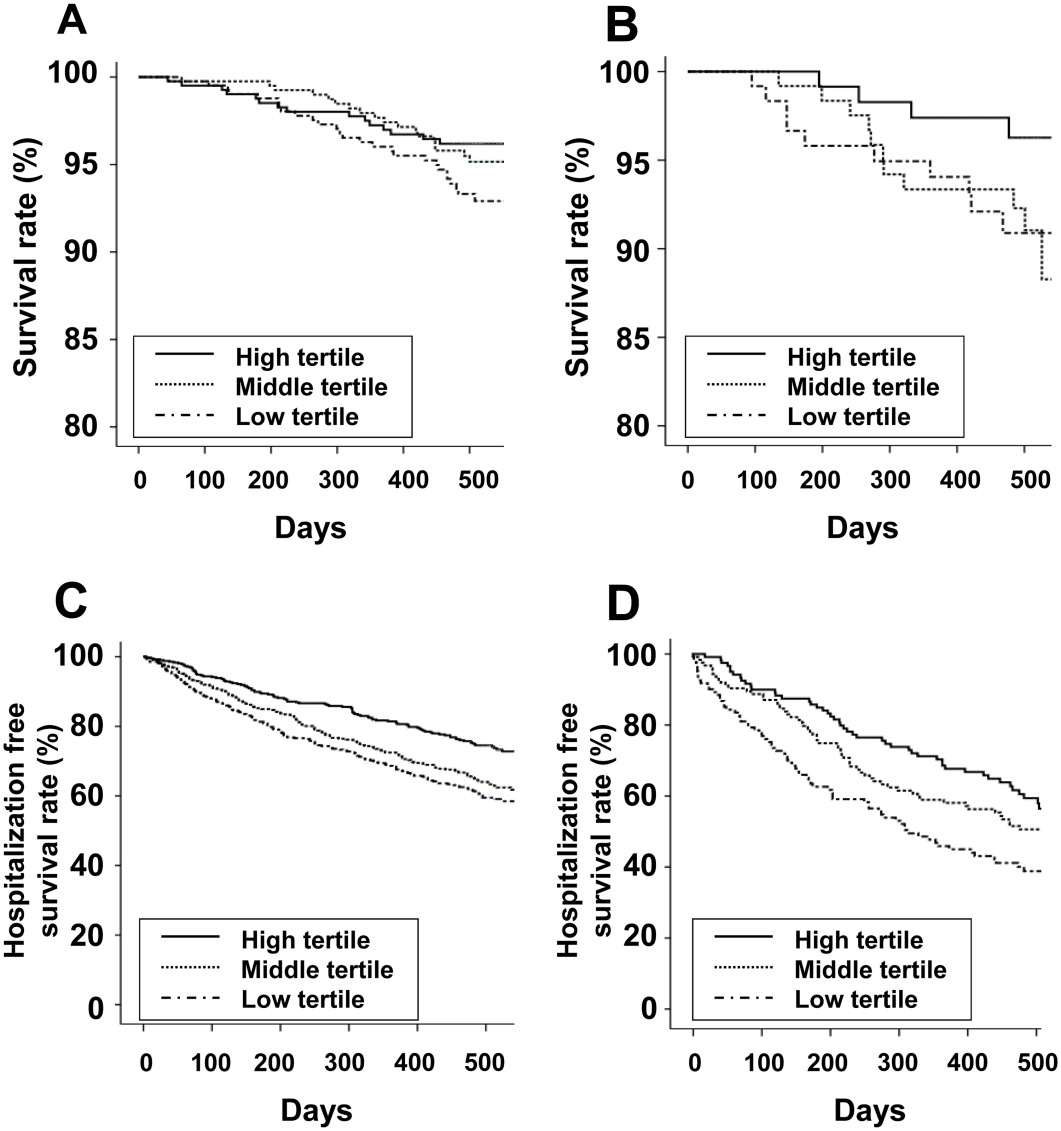
Kaplan-Meier curves for patient survival (A, hemodialysis; B, peritoneal dialysis) and hospitalization-free survival (C, hemodialysis; D, peritoneal dialysis) among the mental component summary score tertiles. (A) Survival rate of hemodialysis patients (High tertile: 99.5% at 100 days and 96.2% at 500 days; Middle tertile: 99.8% at 100 days and 95.2% at 500 days; Low tertile: 99.8% at 100 days and 93.3% at 500 days; *P* = 0.101). (B) Survival rate of peritoneal dialysis patients (High tertile: 100% at 100 days and 96.3% at 500 days; Middle tertile: 100% at 100 days and 92.3% at 500 days; Low tertile: 99.2% at 100 days and 90.9% at 500 days; *P* = 0.169). (C) Hospitalization-free survival rate of hemodialysis patients (High tertile: 94.1% at 100 days and 74.5% at 500 days; Middle tertile: 91.5% at 100 days and 64.0% at 500 days; Low tertile: 88.0% at 100 days and 59.5% at 500 days; *P* < 0.001). (D) Hospitalization-free survival rate of peritoneal dialysis patients (High tertile: 90.0% at 100 days and 59.4% at 500 days; Middle tertile: 88.7% at 100 days and 50.6% at 500 days; Low tertile: 76.9% at 100 days and 38.8% at 500 days; *P* = 0.003).

The follow-up durations (mean ± SD) in HD and PD patients were 489 ± 116 and 467 ± 104 days, respectively. The number of deaths during follow-up was 61 (4.9%) in HD patients and 25 (6.8%) in PD patients. The numbers of participants hospitalized during follow-up was 415 (33.2%) in HD patients and 181 (49.5%) in PD patients. The participants were divided into three groups based on the tertile values for MCS or PCS. Kaplan–Meier analyses showed that both HD and PD patients with a low tertile for PCS had poor patient survival and poor first hospitalization-free survival (Fig 1A–1D). Both HD and PD patients with a low tertile for MCS had poor first hospitalization–free survival (Fig 2A–2D).

Univariate Cox regression analysis showed that an increased PCS in both HD and PD patients was positively associated with patient survival and first hospitalization–free survival (Table 4). An increased MCS in both HD and PD patients was positively associated with first hospitalization–free survival only. Multivariate Cox regression analysis showed that an increased PCS or MCS in HD patients was positively associated with first hospitalization–free survival only. However, an increased PCS or MCS in PD patients was positively associated with patient survival and first hospitalization–free survival.

**Table 4:** Cox regression analyses according to quality-of-life scale scores.

	Univariate		Multivariate	
Patient survival	Hazard ratio (95% CI)	*P*-value	Hazard ratio (95% CI)	*P*-value
HD				
PCS	0.982 (0.971–0.993)	0.001	0.989 (0.977–1.000)	0.055
MCS	0.992 (0.981–1.004)	0.173	0.996 (0.984–1.008)	0.492
PD				
PCS	0.972 (0.955–0.990)	0.002	0.975 (0.956–0.995)	0.016
MCS	0.982 (0.964–1.000)	0.054	0.978 (0.958–0.999)	0.037
**First HFS**				
HD				
PCS	0.984 (0.980–0.989)	0.000	0.998 (0.983–0.992)	0.000
MCS	0.990 (0.986–0.994)	0.000	0.992 (0.987–0.997)	0.001
PD				
PCS	0.984 (0.977–0.991)	0.000	0.987 (0.979–0.994)	0.001
MCS	0.988 (0.980–0.995)	0.001	0.987 (0.980–0.994)	0.001

Multivariate analysis was performed by using enter mode with adjustment for age, sex, body mass index, education level, dialysis vintage, diabetes mellitus, cerebrovascular disease, coronary artery disease, serum albumin level, blood urea nitrogen level, serum creatinine level, intact parathyroid hormone level, and total cholesterol level.

Abbreviations: CI, confidence interval; HD, hemodialysis; PCS, physical component scale; MCS, mental component scale; HFS, hospitalization-free survival.

### Clinical outcomes according to frailty and disability

In HD patients, univariate and multivariate Cox regression analyses showed that the presence of frailty or disability was inversely associated with patient survival and first hospitalization-free survival (Table 5). In PD patients, the presence of frailty was inversely associated with first hospitalization-free survival in both univariate and multivariate analyses. The presence of disability was inversely associated with patient survival only in univariate analysis.

**Table 5:** Cox regression analyses according to frailty or disability.

	Univariate		Multivariate	
Patient survival	Hazard ratio (95% CI)	*P*-value	Hazard ratio (95% CI)	*P*-value
HD				
Frailty	3.069 (1.836–5.130)	0.000	2.352 (1.364–4.056)	0.002
Disability	2.940 (1.733–4.988)	0.000	2.136 (1.206–3.783)	0.009
PD				
Frailty	2.654 (1.192–5.910)	0.017	1.754 (0.683–4.499)	0.243
Disability	1.391 (0.615–3.148)	0.428	0.974 (0.401–2.363)	0.953
**First HFS**				
HD				
Frailty	1.871 (1.541–2.271)	0.000	1.558 (1.265–1.919)	0.000
Disability	1.712 (1.354–2.165)	0.000	1.431 (1.116–1.836)	0.005
PD				
Frailty	1.782 (1.330–2.389)	0.000	1.405 (1.017–1.940)	0.039
Disability	1.284 (0.941–1.753)	0.115	1.160 (0.837–1.606)	0.372

Multivariate analysis was performed by using enter mode with adjustment for age, sex, body mass index, education level, dialysis vintage, diabetes mellitus, cerebrovascular disease, coronary artery disease, serum albumin level, blood urea nitrogen level, serum creatinine level, intact parathyroid hormone level, and total cholesterol level. Abbreviations: CI, confidence interval; HD, hemodialysis; PCS, physical component scale; MCS, mental component scale; HFS, hospitalization-free survival.

## Discussion

Our study included only relatively stable maintenance dialysis patients. There was no significant difference in frailty phenotypes between the two modalities; however, disability was more common in PD patients than in HD patients. MCS and PCS were more favorable in HD patients than in PD patients. Among the kidney disease–specific scales, symptom/problems, sleep, quality of social interaction, and social support were more favorable in HD patients than in PD patients, whereas patient satisfaction and dialysis staff encouragement were more favorable in PD patients than in HD patients. PCS was associated with patient survival and hospitalization, whereas MCS was associated with hospitalization only.

Although many studies showed similar HRQoL results between HD and PD patients, some studies showed better outcomes in HD patients. Wu et al. investigated the difference in HRQoL between HD and PD patients [24]. They evaluated the HRQoL at baseline and at 1 year after dialysis initiation. The HRQoL scale scores were similar between the two modalities; however, the HRQoL improvement during the 1-year period was greater in HD patients than in PD patients. A study in Brazil showed that the functional capacity, physical aspect, and social aspect were better in HD patients than in PD patients [10]. A review that included 26 studies showed that the baseline HRQoL scale scores were mostly higher in PD patients than in HD patients [25]. However, the follow-up mental health scale scores were comparable between the two modalities, and the follow-up physical scale scores were relatively better in HD patients than in PD patients. In our study, four scales among eight items in SF-36, the OHR, the PCS, and the MCS were better in HD patients than in PD patients. Symptom/problems, sleep, quality of social interaction, and social support among kidney disease-specific items were better in HD patients than in PD patients. In Korea, the proportions of patients undergoing HD, kidney transplantation, and PD in 2014 were 71.0%, 19.8%, and 9.2%, respectively [2]. PD is mostly offered only at tertiary centers, whereas HD is offered in both tertiary and non-tertiary centers [2]. Therefore, HD is considered the first choice among renal replacement therapies in most centers. In Korea, when ESRD patients have contraindications for HD, such as poor vascular status, intractable intradialytic hypotension, or a preference for PD, PD may be recommended. In addition, in our study, all the tertiary centers were located within the Daegu metropolitan area, whereas the HD centers were distributed between both the Daegu metropolitan area and Kyungsangpook-do. Thus, some PD patients must travel to another area to visit the hospital. These points may be associated with the favorable SF-36 results of the HD patients.

In the present study, the scale scores for dialysis care–related patient satisfaction and dialysis staff encouragement were higher in PD patients than in HD patients. These results were similar to those of previous studies [17,26–29]. PD patients were mostly followed at tertiary centers, which may provide more information about the different modalities. In addition, PD patients were three times more likely to switch to HD, often for various causes, whereas dialysis staff in tertiary centers may encourage patients to maintain their current modality [30]. These factors may be associated with the increased scores of PD patients in patient satisfaction and dialysis staff encouragement. Juergensen et al. showed that PD patients were more satisfied with their overall care and perceived less disturbance in their lives than did HD patients [27]. A national multicenter study compared patient satisfaction by using three overall ratings and 20 items rating specific aspects of dialysis care between HD and PD [28]. All ratings or items were greater in PD patients than in HD patients. A previous study with KDQOL–SF 1.3 also showed better patient satisfaction and dialysis staff encouragement in PD patients than in HD patients; however, statistical significance was only observed in patient satisfaction [29].

The association between HRQoL and clinical outcomes in HD patients is well-known [11–13]. Another study investigated the association between the two variables in ESRD patients but did not perform subgroup analysis according to dialysis modality [31]. Other studies evaluated the association between the two variables in PD patients but used only SF-36 items [32,33]. The present study investigated KDQOL–SF including kidney disease–specific items and performed subgroup analyses of each dialysis modality. In our study, PCS was associated with patient survival and hospitalization, but MCS was associated with hospitalization only. We enrolled relatively stable maintenance dialysis patients with a high survival rate. Concerning the shape of the Kaplan–Meier curve, long-term follow-up may result in statistical significance.

In our study, the differences in frailty and disability between the two dialysis modalities are important findings. Disability is closely associated with frailty; however, the present study showed no significant difference in frailty phenotype between the two modalities; however, the presence and number of disability components were higher in PD patients than in HD patients. These results reveal that a greater proportion of PD patients than HD patients had a disability regardless of physical functional status. Disability is defined as difficulty with ADLs, which are mainly performed outside of the dialysis period. PD patients receive the dialysate during an entire day, which can lead to disability. If the peritoneal dialysate is associated with disability, night intermittent PD may improve this problem. In our study, the type of PD was not evaluated; however, <20% of total PD patients in Korea receive automated PD [34]. Most PD patients in our study mainly received continuous ambulatory PD.

The BMI, serum albumin level, and serum creatinine level, which are well-known nutritional markers, differed between HD and PD patients in our study. In ESRD patients, malnutrition is closely associated with sarcopenia and quality of life, and these differences can indirectly influence HRQoL or frailty. In the present study, BMI was greater in PD patients than in HD patients, but serum albumin level was greater in HD patients than in PD patients. However, the differences in these two variables can be associated with overhydration. PD patients are generally overhydrated compared with HD patients [35]. An overhydrated volume status leads to overestimation of body weight, which results in increased BMI, and dilution of serum albumin, which results in a decreased serum albumin level [36]. In addition, the serum creatinine level was greater in PD patients than in HD patients. Serum creatinine is another classic marker of muscle mass in the general population; however, the association between serum creatinine level and muscle mass is very complex in ESRD patients. Serum creatinine levels are dependent on various factors such as age, sex, race, residual renal function, dialysis adequacy, and comorbidities. The association between these variables and nutritional status should be carefully interpreted in ESRD patients, as a previous study showed no association between serum creatinine level and muscle mass [37]. To overcome these differences, we analyzed HRQoL or disability by using multivariate analyses adjusted to these variables, and our results showed that the dialysis modality is associated with the HRQoL scales.

Frailty, disability, and PCS would be interchangeably to identify decreased physical functional status, but they are distinct clinical entities. These three concepts can influence each other. The associations among these indicators are very complex and it is difficult to identify causal relationships. It may be difficult to identify superiority among these measurements, but, for specific populations or outcomes, one may be superior to the others. Our study showed that in HD patients, PCS was only positively associated with first hospitalization–free survival, but the presence of frailty or disability was inversely associated with both patient survival and first hospitalization-free survival. In PD patients, PCS was positively associated with both patient survival and first hospitalization–free survival. However, the presence of frailty was only inversely associated with first hospitalization-free survival, and the presence of disability was not associated with the two clinical outcomes. Our results revealed that the predictive value of frailty or disability for clinical outcomes may be superior to that of PCS in HD patients, and the predictive value of PCS for clinical outcomes may be superior to that of frailty or disability in PD patients. Further investigation is needed to identify superiority among these measurements in dialysis patients.

The present study has a few limitations. First, it is limited by its retrospective nature, as a post-hoc analysis of participants enrolled in a previous study [18]. Second, we did not evaluate frailty phenotype by using physical performance-based measurements; rather, it was assessed with a self-reported questionnaire only. This may be associated with inaccuracies in frailty criteria. Third, information about dialysis adequacy or additional dialysis modalities such as hemodiafiltration or automated PD was not collected in our study. However, previous studies did not show significant differences between automated PD and continuous ambulatory PD, or between hemodiafiltration and HD [38–42]. Dialysis adequacy also influences clinical outcomes. However, Kt/V in a Korean registry was 1.55 ± 0.30 in HD patients [43]. In Korea, most patients receive adequate dialysis. In addition, BUN values were averaged using three different midweek predialysis levels. Averaged predialysis BUN is not a direct marker of dialysis adequacy, because it does not take into account protein intake and catabolism [44]. However, there may be a positive correlation between averaged predialysis BUN level and dialysis adequacy [44]. Fourth, we did not perform validations of disability and frailty questionnaires. Previous studies showed the association between disability or frailty questionnaires and clinical outcomes in various populations. However, few studies performed validation of disability and frailty questionnaires in the Korean population. Park et al. had performed validation for ADL components in the Korean elderly population, and showed their method as an acceptable tool for quantifying disability [45]. To our knowledge, there is no study involving validation of a frailty questionnaire for the Korean population. Fifth, the exclusion of patients with a history of recent hospitalization is associated with selection bias, and more HD patients than PD patients may have been excluded. The population in our study included relatively stable maintenance dialysis patients, and we did not collect data for excluded participants. However, recent hospitalization may be temporarily associated with decreased HRQoL and increased disability or frailty. These factors would result in additional bias. A future prospective multi-ethnic study that includes additional parameters such as dialysis modalities, physical performance–based measurements, or data on reliability and validity is warranted to overcome these limitations.

In conclusion, there was no significant difference in frailty between patients treated with the two dialysis modalities; however, disability was more common in PD patients than in HD patients. Among the SF-36 items, MCS and PCS were more favorable in HD patients than in PD patients. Among the kidney disease-targeted scales, symptom/problems, sleep, quality of social interaction, and social support were more favorable in HD patients than in PD patients; however, patient satisfaction and dialysis staff encouragement were more favorable in PD patients than in HD patients.

## Supporting information

S1 Table Comparison of quality-of-life scores between participants receiving dialysis in a tertiary center and those receiving dialysis in a non-tertiary center. (DOCX)
