# Supplementary material for: Effect of dialysis modality on frailty phenotype, disability, and health-related quality of life in maintenance dialysis patients
Source: PLoS One. 2017 May 3;12(5):e0176814. doi: 10.1371/journal.pone.0176814 (PMC5415130; doi:10.1371/journal.pone.0176814)
Supplement: S1 Table — (DOCX) [file pone.0176814.s001.docx]

| **Short Form-36 scale** |  | **Tertiary HD**  **(n = 393)** | **Non-tertiary HD**  **(n = 343)** | **Tertiary PD**  **(n = 857)** | ***P*-value** |
| --- | --- | --- | --- | --- | --- |
| PF |  | 74.9 ± 25.8 | 74.7 ± 23.3 | 72.7 ± 2.3 | 0.346 |
| RP |  | 63.4 ± 42.6 | 66.7 ± 40.9 | 61 ± 41.1 | 0.074 |
| BP |  | 78.6 ± 25.6 | 77.6 ± 25.8 | 76.3 ± 24.7 | 0.466 |
| GH |  | 46.7 ± 20.5 | 44.5 ± 21.9 | 41.2 ± 20.5* | 0.004 |
| VT |  | 45.1 ± 20.5 | 44.6 ± 21.9 | 44.5 ± 20.5 | 0.908 |
| SF |  | 75.6 ±30.2 | 76.3 ± 27.1 | 73.0 ± 26.4 | 0.165 |
| RE |  | 71.4 ± 42.1 | 73.2 ± 40.6 | 64.1 ± 43.1*^#^ | 0.002 |
| MH |  | 58.4 ± 19.1 | 59.4 ± 20.7 | 58.2 ± 20.5 | 0.530 |
| OHR |  | 42.2 ± 26.1 | 36.9 ± 26.3* | 33.5 ± 24.5*^#^ | <0.001 |
| PCS |  | 61.8 ± 21.0 | 61.6 ± 19.9 | 59.1 ± 20.6 | 0.127 |
| MCS |  | 59.4 ± 20.2 | 59.6 ± 20.7 | 56.2 ± 20.2* | 0.025 |
| **KD-specific scale** |  |  |  |  |  |
| Symptom/problems |  | 81.0 ± 13.3 | 80.8 ± 15.0 | 77.4 ± 16.3*^#^ | 0.001 |
| Effects of KD |  | 74.2 ± 19.1 | 71.7 ± 19.3 | 74.6 ± 18.7 | 0.030 |
| Burden of KD |  | 36.7 ± 26.3 | 33.6 ± 26.5 | 36.3 ± 26.1 | 0.089 |
| Work status |  | 20.9 ± 33.5 | 31.0 ± 36.6* | 27.7 ± 37.5* | <0.001 |
| Cognitive function |  | 86.4 ± 17.7 | 86.1 ± 17.2 | 85.2 ± 17.6 | 0.630 |
| Quality of social interaction |  | 76.3 ± 22.2 | 76.2 ± 21.6 | 72.3 ± 21.7*^#^ | 0.012 |
| Sexual function |  | 80.6 ± 24.7 | 77.0 ± 26.4 | 74.8 ± 27.1 | 0.363 |
| Sleep |  | 64.4 ± 22.6 | 64.1 ± 20.9 | 63.0 ± 20.8 | 0.626 |
| Social support |  | 74.7 ± 26.9 | 65.5 ± 26.6* | 65.0 ± 27.4* | <0.001 |
| Patient satisfaction |  | 69.1 ± 24.5 | 62.5 ± 21.5* | 70.5 ± 23.1^#^ | <0.001 |
| DSE |  | 86.3 ± 19.1 | 84.5 ± 18.8 | 90.5 ± 15.8*^#^ | <0.001 |
